# The Role of Hematological Indices in Patients with Acute Coronary Syndrome

**DOI:** 10.1155/2017/3041565

**Published:** 2017-10-03

**Authors:** Jan Budzianowski, Konrad Pieszko, Paweł Burchardt, Janusz Rzeźniczak, Jarosław Hiczkiewicz

**Affiliations:** ^1^Department of Cardiology, Hospital Nowa Sól, Nowa Sól, Poland; ^2^Department of Biology and Environmental Sciences, Poznań University of Medical Sciences, Poznań, Poland; ^3^Department of Cardiology, J. Struś Hospital, Poznań, Poland

## Abstract

An increased systemic and local inflammation plays a key role in the pathophysiology of acute coronary syndrome (ACS). This review will discuss the role of hematological indices: white blood cells (WBC), neutrophil to lymphocyte ratio (NLR), red cell distribution width (RDW), and platelet indices, that is, platelet to lymphocyte ratio (PLR), mean platelet volume (MPV), and platelet distribution width (PDW) in the case of ACS. In recent years, a strong interest has been drawn to these indices, given that they may provide independent information on pathophysiology, risk stratification, and optimal management. Their low-cost and consequent wide and easy availability in daily clinical practice have made them very popular in the laboratory testing. Furthermore, many studies have pointed at their effective prognostic value in all-cause mortality, major cardiovascular events, stent thrombosis, arrhythmias, and myocardial perfusion disorders in terms of acute myocardial infarction and unstable angina. The most recent research also emphasizes their significant value in the combined analysis with other markers, such as troponin, or with GRACE, SYNTAX, and TIMI scores, which improve risk stratification and diagnosis in ACS patients.

## 1. Introduction

Coronary heart disease (CHD), most commonly caused by atherosclerosis, is the leading cause of death worldwide. Atherosclerosis is a systemic, lipid-driven immune inflammatory disease [1]. Inflammation, one of the factors leading to coronary artery disease (CAD), can be not only local but also systemic. Research carried out by Dutta et al. [2] proved that myocardial infarction is linked to an increased myeloid activity. Interestingly, it has also been shown that in the case of mice with an induced myocardial infarction, the sympathetic nervous system (SNS) becomes activated. This, in turn, induces the release of hematopoietic stem cells (HSPCs) from bone marrow niches, which consequently causes the further systemic stimulation of atherosclerotic plaques.

The chronic low-grade inflammation plays a key role in the initiation and development of the atherosclerotic plaque, which subsequently leads to the plaque's instability with a thrombus formation. Inflammation is also considered to be one of the main causes of diabetes, hyperlipidemia, metabolic syndrome, and endothelial dysfunction [3]. The inflammation leading to ACS encourages research into the clinical usage of new inflammatory biomarkers.

In this review, we shall describe the main hematological indices and their prognostic and diagnostic value in patients with ACS. In recent years, strong interest has arisen in these indices, given that they may provide independent information on pathophysiology, risk stratification, and optimal management.

The main advantage of hematological indices is that they are relatively inexpensive and thus widely and easily available in daily clinical practice. They have also proven their diagnostic and prognostic value in many cardiovascular diseases including CAD, atrial fibrillation following the coronary artery bypass graft (CABG) procedure, acute and chronic cardiac insufficiency, cardiac arrhythmias, and pulmonary hypertension.

## 2. White Blood Cell Count (WBC)

Leukocytes play a key role in the pathophysiology of ACS, given their effect on the instability of atherosclerotic plaques. In the initial stage, leukocytes permeate endothelial cells and become activated when reaching the tunica intima. They induce the formation of microvascularity there and, as a result, make plaques more susceptible to rupture [4]. Many studies have indicated that leukocytosis is related to an increased cardiovascular mortality rate. What is more, leukocytosis also proved to be of prognostic value when assessing adverse clinical outcomes [5–7].

In the study of Sabatine et al., the elevated WBC count was found to be a relevant death risk factor during the first 30 days and 6 months following the myocardial infarction among patients with ACS (UA, NSTEMI). Furthermore, the elevated level of WBC was also related to a more advanced CAD as well as epicardial and myocardial perfusion disorders [8]. In another study, the WBC > 10,000 pointed to increased mortality among AMI and UA patients. [9]

Many prospective studies have shown that the increased concentration of leukocytes on admission was connected not only to the development of worse microvascular injury, congestive heart failure, and shock but also to the elevated mortality rate in patients with ACS [10].

## 3. Neutrophil to Lymphocyte Ratio (NLR)

NLR is easily measured by dividing neutrophil count by lymphocyte count in a differential white blood cells (WBC) sample. It is one of the best-assessed hematological biomarkers, which provides prognostic and diagnostic information in ACS. Its role in cardiovascular diseases has been studied extensively in the past few years [11, 12].

The study of Sezer et al. proved that the increased number of neutrophils and MPV in patients with a front wall myocardial infarction is strongly and independently connected to the development of microvascular reperfusion injury after recanalisation of infarct-related artery [13]. In another study, activated neutrophils called polymorphonuclear cells (PMN) were found in coronary thrombi in patients with myocardial infarction who were undergoing primary percutaneous coronary intervention (PCI). PMN release neutrophil extracellular traps (NETs) at the culprit lesion site. NETs are highly proinflammatory and prothrombotic fibers which can entrap leucocytes and propagate thrombosis. NETs proved to be correlated negatively with ST-segment resolution (STR) and positively with infarct size [14]. By contrast, lymphocytes, especially B2 and T helper, as the elements of the adaptive immune system, could mute and limit inflammation. The lower lymphocyte counts were associated with atherosclerosis progression and adverse clinical outcomes in patients with heart failure and ACS [15–17].

The combination of neutrophil and lymphocyte parameters has a better prognostic value than each parameter separately [18]. Kalay et al. demonstrated that NLR is related to the progression of coronary atherosclerosis, the process which is a strong and independent predictor of future coronary events [19]. In Wang et al. meta-analysis, NLR was a predictor of all-cause mortality and cardiovascular events in patients undergoing angiography or cardiac revascularization [20].

In the study of Tamhane et al., the admission NLR was described as a predictor of in-hospital and 6-month mortality in patients who undergo PCI. In the same study, it was proved that higher NLR was associated with diabetes and heart failure [21].

In recent years, numerous papers have been published regarding the value of NLR in predicting short- and long-term mortality in patients with ST-segment elevation (STEMI) [22–25] and with non-NSTEMI [26]. Preprocedural elevated NLR was also linked to an increased risk of significant ventricular arrhythmias during PCI [27].

NRL enables a clinician to predict stent thrombosis and the high mortality rate among patients with STEMI. NLR > 4.9 had 70% accuracy and 65% specificity in predicting in-hospital mortality. In a multidimensional analysis, NLR was strongly linked to stent thrombosis [28].

Furthermore, NRL itself is referred to the complexity and severity of ACS assessed by SYNTAX score, GRACE scale, and TIMI score [29–31].

## 4. Red Cell Distribution Width (RDW)

RDW which is a part of a standard complete blood count (CBC) is a measure of variations in the volume of red blood cells. An elevation in RDW is known as anisocytosis. An increased level of RDW has been found in patients with vitamin B12, iron, and folate deficiency. RDW has also been observed after blood transfusion and hemolysis [32].

In the study of Patel et al., the RDW values above 14.0% were significantly related to a decreased red blood cell deformability, which can impair the blood flow through microcirculation. The resultant diminution of oxygen supply at the tissue level may help to explain the increased risk of adverse cardiovascular events associated with elevated RDW [33]. In 2007, Felker was one of the first authors who proved that the elevated RDW is a useful biomarker of morbidity and mortality among patients with heart failure [34]. In the study of Arbel et al., the RDW level of 12% and above is associated with an increased risk of cardiovascular morbidity and all-cause mortality in both anemic and nonanemic patients [35].

Many studies have highlighted that the increased RDW has also been linked to peripheral artery disease (PAD) [36], chronic obstructive pulmonary disease (COPD) [37], renal failure [38], sepsis and shock sepsis [39], cerebral atherosclerosis [40], stroke [41], and pulmonary hypertension [42]. Tonelli et al. indicated a relationship between higher levels of RDW and the risk of death and adverse cardiovascular outcomes in people with prior myocardial infarction but without symptomatic heart failure [43]. Moreover, it was shown that the elevated RDW was connected to a higher mortality rate in patients with a myocardial infarction (with or without anemia) [44–47].

In their study, Lippi et al. showed that the combined measure of RDW and troponin T (cTnT) increased diagnostic sensitivity to 99%, which meant that the combined measure was more effective in diagnosing ACS than the measure of cTnT alone [48]. Moreover, it was proved that RDW is an essential predictor of CAD severity among patients with acute myocardial infarction (AMI) [49].

## 5. Platelet Indices: PLR, PDW, and MPV

Regardless of their role in the general (systemic) inflammatory response, platelets have been closely related to the activation and coordination of endothelium. It has recently been observed that there is a close relation between cardiovascular mortality and the number of platelets or their ability to aggregate. Platelets play a key role in the pathophysiology of ACS. Compounded with fibrin, platelets form coronary thrombus [1]. The CADILLAC study has shown that the level of platelets (which does not affect the effectiveness of percutaneous interventions) is significantly correlated with the incidence of restenosis and stent thrombosis [50], given the function of platelets in the local as well as general inflammatory response and their aspirin resistance [51, 52].

Platelets participate in the creation of blood clots and deliver mediators which develop and sustain a local inflammatory response [53]. MPV and PDW are important and simple markers which significantly increase during platelet activation [54]. Furthermore, these indices are helpful in the evaluation of thromboembolic diseases.

## 6. Platelet to Lymphocyte Ratio (PLR)

It turns out that the platelet to lymphocyte ratio is a useful parameter describing the systemic inflammatory response. Thus, it has become an important prognostic factor in numerous diseases. It has been shown that PLR correlates with the prognosis in esophageal, ovarian, rectal, and hepatocellular carcinoma as well as glioma multiform [55].

The roles of PLR and other complex markers of systemic inflammatory response have been primarily described in relation to the prognosis of ACS. It has been shown that PLR correlates with a greater overall mortality in patients with NSTEMI [56]. In the recently published (prospective) study involving 5886 patients, the same relation for STEMI has been presented [57]. The same study also showed that high PLR correlates with the recurrence of myocardial infarction, stroke, and subsequent heart failure. It seems that PLR is also helpful in predicting long-term results of percutaneous interventions and it can help select patients with a higher risk of no-reflow syndrome after pPCI [58, 59].

## 7. Platelet Distribution Width (PDW)

Platelet distribution width (PDW) indicates a varied size of platelets. The number of large immature platelets in patients with ACS is caused by an increased bone marrow activity during the process known as thrombocytopoiesis.

PDW measured on admission is a cheap and generally available biomarker which allows for predicting the development of heart failure in patients with ACS after PCI [60]. Bekler et al. showed that an increased level of PDW (>17%) was related to the severity of CAD in patients with ACS. In the same study, an elevated PDW, diabetes mellitus, and myocardial infarction (MI) were positively correlated with a high Gensini score [61]. In a different study, PDW was greater in patients with STEMI than in those with stable CAD [62]. PDW also serves as a useful prognostic factor for long-term mortality in patients after AMI [63, 64].

## 8. Mean Platelet Volume (MPV)

MPV is a useful, indirect, and easily marked biomarker of platelet activity. Numerous studies support the association of MPV with adverse cardiac outcomes in patients with ACS.

MPV was a strong and independent predictor of impaired reperfusion and 6-month mortality in STEMI patients who underwent PCI [65, 66]. A similar correlation was found in NSTEMI patients [67, 68]. Moreover, Chu et al. showed that in patients who underwent PCI, the elevated MPV occurred in patients who developed restenosis [69]. Similarly, Huczek et al. proved that MPV was significantly higher in patients with ACS who developed an early stent thrombosis. It correlated with a poor dual antiplatelet responsiveness [70]. In another study of 462 patients with CAD who underwent PCI, higher MPV levels were independently associated with high residual platelet reactivity after both aspirin and clopidogrel treatments [71]. This is due to the fact that larger platelets are more often reticulated than smaller platelets containing more prothrombotic material (thromboxane A2, platelet factor 4, alpha-granules, P-selectin, and platelet-derived growth factor), which is an independent predictor of a poor response to dual antiplatelet therapy [72].

MPV turned to be independently responsible for the slow coronary flow (SCF) occurrence and its extent [73]. In the recent years, the correlation of WBC to MPV has been named as the WBC/MPV ratio (WMR). The relationship between WMR and major adverse cardiovascular events (MACE) in patients with NSTEMI [74] and STEMI [75] was more prominent than with WBC and MPV, respectively.

## 9. Conclusions

There is a high demand for a reliable, accessible, noninvasive, and hematological prognostic marker in ACS, which would identify patients of high cardiovascular risk in secondary prevention and tailor the therapy to their needs. Many of the indices presented here reflect the complex pathophysiology of ACS. The inflammatory processes play a key role in the development of atherosclerosis, destabilisation of atherosclerotic plaques and formation of clots on the plaque surface [76]. The significance of NLR, PLR, PDW, MPV, and RDW in the prognosis of ACS has been indicated in many studies as it has been shown above. The most crucial studies concerning hematological indices have been summarized in Table 1.

Dutta et al. showed that ACS increases inflammation in the atherosclerotic plaques within months [2]. Therefore, the questions to be asked are at which stage of ACS would it be best to test biomarkers and would repeated tests improve their prognostic value? It is worth emphasizing that EDTA as an anticoagulant can cause platelet swelling thereby affecting the value of PDW and MPV [77]. Moreover, it is worth paying attention to the possible influence of medication on the value of hematological biomarkers; for example, statins had an effect on the higher value of PDW [78]. In a different study, statins considerably diminished MPV [79]. It is also important to mention that the value of hematological biomarkers in ACS patients is affected by other health disorders such as chronic renal failure, anemia, thrombocytopenia, thyroid dysfunction, dyslipidemia, diabetes, and hypertension. What is more, these disorders anticipate worse prognosis for patients with ACS, given their increased chance of heightened inflammation, oxidative stress, and apoptosis in bone marrow, which all negatively affect the function of erythropoiesis (inflammatory cytokines suppress the maturation of erythrocytes). Interestingly, biomarkers have an additional prognostic value for ACS patients if they are analysed with other inflammatory markers (such as CRP and fibrinogen) or with GRACE, SYNTAX, and TIMI risk scores [80–84].

## Figures and Tables

**Table 1:** Summary of some studies investigating diagnostic and prognostic role of the most important hematological indices.

Study type	Study	Sample size	Main findings	References
*WBC*				
Retrospective	Cannon et al. (2001)	7651 patients with ACS	WBC count of >10,000 was associated with increased 30-day and 10-month mortality.	[9]
Retrospective	Barron et al. (2000)	975 patients with MI	Elevation in WBC count was associated with reduced epicardial blood flow and myocardial perfusion, thromboresistance, and a higher incidence of new congestive heart failure and death.	[10]
Prospective	Sabatine et al. (2002)	2220 patients with UA/NSTEMI	Higher baseline WBC count was associated with impaired epicardial and myocardial perfusion, more extensive CAD, and higher six-month mortality rates.	[8]
Retrospective	Gurm et al. (2003)	4450 patients	A low or an elevated preprocedural WBC count in patients undergoing PCI is associated with an increased risk of long-term death.	[11]
Prospective	Chia et al. (2009)	363 patients with STEMI	Elevated leucocyte and neutrophils are predictors of adverse cardiac events.	[12]
*NLR*				
Prospective	Duffy et al. (2006)	1046 patients who underwent PCI	The NLR was an independent significant predictor of long-term mortality in patients who have undergone coronary angiography.	[18]
Prospective	Tamhane et al. (2008)	2833 patients with ACS	NLR was a predictor of in-hospital and 6-month mortality in patients who undergo PCI.	[21]
Prospective	Núñez et al. (2008)	515 patients with STEMI	NLR was a useful marker to predict subsequent mortality in patients admitted for STEMI, with a superior discriminative ability than total WBC.	[22]
Prospective	Azab et al. (2010)	1345 patients with NSTEMI	NLR is an independent predictor of short-term and long-term mortalities in patients with NSTEMI.	[26]
Retrospective	Chatterjee et al. (2011)	30,798 records who have undergone coronary angiography	A preprocedural NLR, elevated WBC count, and neutrophils were predictors of significant ventricular arrhythmias in patients undergoing PCI.	[27]
Prospective	Akpek et al. (2012)	418 patients with STEMI who underwent PCI	The NLR was independently associated with the development of no-reflow and in-hospital MACEs in patients with ST-segment elevation myocardial infarction undergoing primary PCI.	[23]
Prospective	Sahin et al. (2013)	840 patients with STEMI who underwent PCI	NLR was the independent predictor for SYNTAX score in patients with STEMI.	[24]
Retrospective	Sawant et al. (2014)	250 consecutive STEMI patients	NLR based on an optimal cut-off value of 7.4 was an excellent predictor of short- and long-term survival in patients with revascularized STEMI.	[31]
Retrospective	Ayça et al. (2015)	102 patients with stent tshrombosis and 450 patients with STEMI	In patients with STEMI, preprocedural high NLR was associated with both stent thrombosis and higher mortality rates.	[28]
Prospective	Yaylak et al. (2016)	A total of 213 subjects with inferior STEMI	NLR was an independent predictor of RVD in patients with inferior STEMI undergoing primary PCI.	[25]
*RDW*				
Retrospective	Nabais et al. (2009)	1796 patients with ACS	There is a graded independent association between higher RDW values and adverse outcomes in patients with ACS.	[45]
Prospective	Lippi et al. (2009)	456 patients with ACS	RDW at admission might be considered with other conventional cardiac markers for the risk stratification of ACS patients admitted to emergency departments.	[48]
Prospective	Dabbah et al. (2010)	1709 patients with AMI	RDW is a predictor of mortality after AMI. Moreover, an increase in RDW during hospitalization also portends adverse clinical outcome.	[44]
Retrospective	Uyarel et al. (2011)	2506 STEMI patients	RDW at admission was a predictor of in-hospital and long-term cardiovascular mortality.	[46]
Prospective	Isik et al. (2012)	135 patients with STEMI	RDW is a marker indicating long-term prognosis.	[47]
Prospective	Timóteo et al. (2015)	787 patients with ACS	Combination of RDW with GRACE score improves the predictive value for all-cause mortality.	[80]
*PLR*				
Observational study	Azab et al. (2012)	619 patients with NSTEMI	PLR is a significant independent predictor of long-term mortality after NSTEMI.	[56]
Prospective	Kurtul et al. (2014)	1016 patients with ACS	PLR at admission is significantly associated with the severity and complexity of coronary atherosclerosis in patients with ACS. Increased PLR is an independent predictor of higher SYNTAX score in patients with ACS who undergo urgent CA.	[82]
Retrospective	Acet et al. (2016)	800 patients with STEMI	PLR, RDW and monocyte were associated with GRACE score in patients with STEMI.	[81]
Retrospective	Yildiz et al. (2015)	287 patients with STEMI	High preprocedural PLR and NLR levels are significant and independent predictors of no-reflow in patients undergoing primary PCI.	[58]
Prospective	Sun et al. (2017)	5886 patients with STEMI	Higher PLR was associated with recurrent myocardial infarction, heart failure, ischemic stroke, and all-cause mortality in patients with STEMI.	[57]
Prospective	Vakili et al. (2017)	215 patients with STEMI	PLR and NLR were associated with no-reflow phenomenon in patients with STEMI treated with pPCI.	[59]
*PDW*				
Prospective	De Luca et al. (2010)	1882 patients undergoing coronary angiography + IMT in 359 patients	PDW is not related to the extent of CAD and carotid IMT. PDW positively correlated with age, weight, waist circumference, and prevalence of diabetes.	[78]
Prospective	Rechciński et al. (2013)	538 patients who underwent primary PCI in acute MI	PDW and P-LCR are prognostic predictors after MI.	[63]
Retrospective	Celik et al. (2015)	306 patients with STEMI	Baseline PDW and MPV are independent correlates of no-reflow and in-hospital MACEs among patients with STEMI undergoing pPCI.	[64]
Retrospective	Bekler et al. (2015)	502 patients with ACS were enrolled.	The group with PDW > 17% had significantly higher Gensini score.	[61]
*MPV*				
Prospective	Huczek et al. (2005)	398 patients with STEMI	MPV is a predictor of impaired reperfusion and mortality in STEMI treated with pPCI.	[65]
Case-control study	Huczek et al. (2010)	36 consecutive ST cases and 72 matched controls	Baseline platelet size is increased in patients with ACS developing early stent thrombosis and correlates with future residual platelet reactivity.	[70]
Systematic review + meta-analisis	Chu et al. 2010	Pooled results from 16 cross-sectional studies involving 2809 patients with CAD	Elevated MPV is associated with AMI, mortality following myocardial infarction, and restenosis following coronary angioplasty.	[69]
Retrospective	Isik et al. (2012)	2467 who underwent coronarography with CAD	Diabetes, smoking, hemoglobin, and MPV were found to be the independent correlates of SCF presence. Moreover, only MPV was identified as an independent correlate of extent of SCF.	[73]
Prospective	Wan et al. (2014)	297 ACS patients	Both MPV and the GRACE score were significant and independent predictors for CVD events. Combination of MPV with the scoring system improved the predictive value.	[83]
Prospective	Niu et al. (2015)	506 ACS patients	Elevated MPV was an independent predictor of 6-month mortality or MI in patients with ACS. The addition of MPV to the GRACE model improved its predictive value.	[84]

ACS: acute coronary syndrome; AMI: acute myocardial infarction; NSTEMI: non-ST elevation myocardial infarction; MI: myocardial infarction; MPV: mean platelet volume; STEMI: ST elevation myocardial infarction; CAD: coronary artery disease; TIMI: thrombolysis in myocardial infarction; WBC: white blood cell count; RDW: red blood cell distribution width; PDW: platelet distribution width; PLR: platelet lymphocyte ratio; P-LCR: platelet large cell ratio; CVD: cardiovascular disease; HF: heart failure; PCI: percutaneous coronary intervention; RVD: right ventricular dysfunction; MACEs: major adverse cardiac events; GRACE: global registry of acute coronary events; SCF: slow coronary flow; IMT: intima media thickness.
